# A DELPHI study priority setting the remaining challenges for the use of routinely collected data in trials: COMORANT-UK

**DOI:** 10.1186/s13063-023-07251-x

**Published:** 2023-03-30

**Authors:** Adam D. N. Williams, Gwyneth Davies, Amanda J. Farrin, Marion Mafham, Michael Robling, Matthew R. Sydes, Fiona V. Lugg-Widger

**Affiliations:** 1 Centre for Trials Research, Cardiff University, Cardiff, UK; 2 UCL Great Ormond Street Institute of Child Health, London, UK; 3 Clinical Trials Research Unit, Leeds Institute of Clinical Trials Research, University of Leeds, Leeds, UK; 4 Clinical Trial Service Unit and Epidemiological Studies Unit, Nuffield Department of Population Health, University of Oxford, Oxford, UK; 5 DECIPHer - Centre for Development, Evaluation, Complexity and Implementation in Public Health Improvement, Cardiff University, Cardiff, UK; 6 MRC Clinical Trials Unit at UCL, Institute of Clinical Trial and Methodology, University College London, London, UK; 7 BHF Data Science Centre, Health Data Research UK, London, UK

**Keywords:** Priority setting, Trials methodology, Routinely collected data, Consensus

## Abstract

**Background:**

Researchers are increasingly seeking to use routinely collected data to support clinical trials. This approach has the potential to transform the way clinical trials are conducted in the future. The availability of routinely collected data for research, whether healthcare or administrative, has increased, and infrastructure funding has enabled much of this. However, challenges remain at all stages of a trial life cycle. This study, COMORANT-UK, aimed to systematically identify, with key stakeholders across the UK, the ongoing challenges related to trials that seek to use routinely collected data.

**Methods:**

This three-step Delphi method consisted of two rounds of anonymous web-based surveys and a virtual consensus meeting. Stakeholders included trialists, data infrastructures, funders of trials, regulators, data providers and the public. Stakeholders identified research questions or challenges that they considered were of particular importance and then selected their top 10 in the second survey. The ranked questions were taken forward to the consensus meeting for discussion with representatives invited from the stakeholder groups.

**Results:**

In the first survey, 66 respondents yielded over 260 questions or challenges. These were thematically grouped and merged into a list of 40 unique questions. Eighty-eight stakeholders then ranked their top ten from the 40 questions in the second survey. The most common 14 questions were brought to the virtual consensus meeting in which stakeholders agreed a top list of seven questions. We report these seven questions which are within the following domains: trial design, Patient and Public Involvement, trial set-up, trial open and trial data. These questions address both evidence gaps (requiring further methodological research) and implementation gaps (requiring training and/or service re-organisation).

**Conclusion:**

This prioritised list of seven questions should inform the direction of future research in this area and should direct efforts to ensure that the benefits in major infrastructure for routinely collected data are achieved and translated. Without this and future work to address these questions, the potential societal benefits of using routinely collected data to help answer important clinical questions will not be realised.

**Supplementary Information:**

The online version contains supplementary material available at 10.1186/s13063-023-07251-x.

## Background

Routinely collected data (RCD) is increasingly being used to support health research, including clinical trials, in the UK and worldwide. RCD include data collected for purposes other than research, including electronic health records (EHR)—the digital version of patient health information (charts, notes, test results) and registry data—a collection of information about individuals, usually focused on a specific diagnosis or condition. The use of RCD in randomised controlled trials has the potential to bring essential benefits to patients participating in clinical trials, healthcare staff supporting clinical trials and members of the public and researchers globally who may be impacted by the trial findings [1].

Accessing data directly from the RCD sources is expected to reduce the burden on patients and care staff otherwise involved in collecting data directly from individuals (bespoke data collection). RCD has the potential to provide data that are more scientifically valid (i.e. long-term data follow-up and high levels of completeness), enhance generalisability (i.e. high retention and more inclusive and diverse sample) and reduce the overall cost to the public of conducting large-scale research studies. Despite growing recognition of such potential benefits of using RCD for trials research, there remain key methodological challenges for researchers with identifying, accessing and using these datasets in practice and several areas of uncertainty [2–6].

Beyond publications of expert opinion and limited examples reporting the (intended) use of RCD for trials, there is very little published evidence to guide researchers in this field. As a result, there remain broad uncertainties over the better approaches to ensure the highest standard of scientific rigour for use of RCD in trials. Anecdotally, these uncertainties include data and linkage quality, access and regulatory compliance, ethical considerations, public involvement and privacy.

Identifying the top uncertainties in this area will direct efforts to ensure that the benefits in major infrastructure for RCD (in particular EHRs) are achieved and translate into benefits for methodology and efficiency in future trials, as well as for patients and their clinical teams. Without this, the potential societal benefits of using RCD to help answer important clinical questions will not be realised. The scope for this work will need to be wide as different countries’ systems and infrastructures vary considerably.

## Methods

### Aim

This study aimed to systematically identify the current methodological research questions and uncertainties related to the use of RCD in trials, from the perspective of relevant stakeholders in the UK.

### Design and setting

We conducted a three-step Delphi method [7, 8] consisting of two rounds of anonymous web-based surveys and a virtual consensus meeting with key UK stakeholders. The particular challenges will vary from country to country, although there will be similarities. The aim of this project was to focus on what can, and should, be done for the UK before moving to a global setting in the future.

### Eligibility

Participants were eligible for the study if they were a UK stakeholder as defined in Table 1 that used RCD for UK-based trials and were 18 + years of age (for public members).

**Table 1:** UK stakeholders included

Stakeholder group	*Definition (examples)*
Trialists/data scientists	*Researchers of all career stages working at academic or private organisations who are involved in the processing of RCD for trials including access, analysis, sharing and archiving*
RCD infrastructures	*Organisations who, as part of their remit, look to improve and develop the use of RCD in trials such as Health Data Research UK*
Funding bodies	*Funders of trials that use RCD (National Institute for Health and Care Research, UK Research and Innovation, Wellcome Trust)*
Data providers	*Registries and RCD providers in the UK, current and future (NHS Digital, SAIL Databank, the electronic Data Research and Innovation Service; eDRIS, Honest Broker Service)*
The public	*Members of the public familiar with the use of RCD in trials (e.g. *via* approval panels, as research partners and public contributors)*
Support networks	*Methodology and clinical trial networks (Trials Methodology Research Partnership, HRB-Trials Methodology Research Network, UK Clinical Research Collaboration)*
Regulating bodies	*Regulators relevant to trials that use RCD (Health Research Authority—Ethics and the Confidentiality Advisory Group, the Medicines and Healthcare products Regulatory Agency, Data Protection Officers, Information Commissioner’s Office)*

### Implementation

#### Survey 1: Identifying all remaining questions and challenges

We used non-probability purposive sampling methods to sample survey respondents [8]. The target population are described in Table 1, and we used the study team’s networks to disseminate the survey to all network members including via newsletters and social media. Stakeholders were sent a link to an online survey page and presented with information about the purpose of the study and how their data will be used. They were asked to tick to confirm consent and then proceed to the survey. Respondents could provide their email addresses to be contacted further by the team regarding the future, second survey, planned consensus meeting and eventual project outputs. That email address was stored in a separate database from the respondent survey responses. We collected limited demographic information on all respondents to assess whether our sampling frame had reached, and the survey been completed by, a range of stakeholders (stakeholder group, type of RCD they worked with/provide/aware of, and country they were based in).

Respondents were asked to supply methodological research questions they felt were of particular importance in relation to RCD for trials. Methodological research questions were clarified as issues that can be answered with research methods and a visual map of the study life cycle was available to prompt respondents to think about all aspects of a research project (without giving specific examples which could have biassed responses) (see Additional file 1). Responses were provided as free-text answers in a field of unconstrained length so respondents could list as many questions as they wished. The survey was live for 9 weeks (January 10 to March 14, 2022). Free-text survey responses were imported into NVivo 12. We used a constant comparative method to identify emergent themes in the survey responses. Questions or comments that were assessed as not relevant were excluded at this stage. This was an iterative and inductive method of reducing the data through constant recoding [9, 10]. This was applied to all survey responses by two researchers [AW, FLW] with input from the wider team as required. This reduced list of questions was then taken forward to the second survey.

#### Survey 2: Selecting the top 10

We used the same sampling method and distribution to networks as survey 1, with the survey open to previous respondents as well as new respondents. The same demographics were collected in addition to asking if respondents had completed the first survey. Respondents were then asked to choose 10 questions they felt were most important for researchers to answer based on their own experiences. Questions were categorised according to their focus in relation to the research process (i.e. trial open, trial data, Public and Patient Involvement). The presentation order was randomly changed for each respondent so that all responses had an equal chance of being selected. A free-text box asked if there were any questions missing for respondents to add in as relevant. The survey was live for 3 weeks (May 2 to May 23, 2022). After the closure of the survey, the questions were then ranked based on their frequency of appearing in the respondent’s top 10. The rankings were reviewed for the whole sample and then separated by stakeholder groups to identify any striking differences between stakeholder groups that may have skewed the whole sample ranking. The highest-ranking questions were taken forward to the consensus meeting, in addition to the responses provided in the free text (i.e. respondents suggested missing questions).

#### Consensus meeting: agreeing the final list

We held a virtual consensus meeting in May 2022. Stakeholders from each group (see list in Table 1) were invited through the completion of the surveys and via professional connections of the authors, with no restrictions placed on the number of attendees from each group. An experienced facilitator moderated the meeting following an agreed agenda. Some authors of this paper attended the meeting, accounting for 40% of the group (6/15). Attendees were not required to provide additional consent to participate in the meeting, and the recording of the meeting was only for the purposes of note-taking. The proposed list of questions was discussed individually, and attendees used Mentimeter® to anonymously vote for items to be included in the final list. Discussions on the final wording of the top list of questions continued by email following the consensus meeting, and the final list was agreed upon in June 2022.

### Data management

Both surveys were developed using Qualtrics, and all responses were collected over encrypted SSL (TLS) connections. All responses were transferred to Cardiff University secure servers and analysed within Nvivo 12 (survey 1) and Microsoft Excel (survey 2). Mentimeter® votes were collected anonymously, and the results were saved on the same university servers.

## Results

### Survey 1: Identifying all remaining questions and challenges

Survey 1 was completed by 66 respondents. The most frequently reported stakeholder group self-identified by respondents was “trialist” (75%). The proportion of respondents from each stakeholder group, geographic spread and type of routinely collected data is shown in Table 2. There were 266 questions or comments reported by respondents. Following thematic analysis, these were grouped into 47 unique questions informed directly by the wording used by respondents. Through review by the wider study team, these were further reduced to a final list of 40 questions for inclusion in the second survey (see Additional file 2). A small number of responses were not included in the second survey as they were deemed to not be relevant to the use of routinely collected data in trials (*n* = 10).

**Table 2:** Survey demographics for respondents to surveys 1 and 2

Question	Survey 1		Survey 2	
	***n* = 66**	**%**	***n* = 88**	**%**
**Stakeholder group**				
I work on trials that use routinely collected data in the UK	51	77	57	65
I am a member of the public familiar with trials that use/seek to use routinely collected data in the UK	6	9	2	2
I/the organisation I work for funds trials that do/could use routinely collected data in the UK	3	5	2	2
I/the organisation I work for supports trials that use/could use routinely collected data in the UK	1	2	13	15
I/the organisation I work for provides/could provide trials with routinely collected data in the UK	4	6	6	7
I/the organisation I work for provides/could provide approval for trials that use routinely collected data in the UK (e.g. MHRA, REC, CAG) | others	1	2	8	9
**What routinely collected data do you already work with?**^**a**^** [multiple choice]**				
Electronic health records [primary care]	33	50	32	36
Electronic health records [secondary care]	56	85	45	51
Mortality data	42	64	40	46
National registries/audits	38	58	25	28
Education	7	11	13	15
Adult social care	6	9	3	3
Children’s social care	8	12	8	9
Criminal justice and benefit	4	6	1	1
Others	1	1	1	1
**What country/ies are you based in?**^**a**^** [multiple choice]**				
Wales	19	29	22	25
England	50	76	55	63
Scotland	12	18	7	8
Northern Ireland	6	9	2	2
Outside the UK	2	3	3	3
**Did you complete the first survey?**				
Yes	N/A	N/A	26	30
Unsure	N/A	N/A	20	23
No	N/A	N/A	40	46
Missing	N/A	N/A	2	2

^a^Multiple responses allowed

### Survey 2: Selecting the top 10

The second survey presented the 40 questions for respondents to select their top 10. There were 88 responses with the majority being trialists (65%), 30% reported completing survey 1, 23% were unsure and 40% had not completed survey 1. Table 2 shows the demographics of respondents from this second survey.

All 40 questions were included in the top 10 of at least 5 respondents, with the highest-ranked question being included by 50 respondents (See Additional file 3). The 14 highest-ranked questions were taken forward to the consensus meeting. These 14 represented the top-ranked questions for all stakeholders. Additionally, three questions included in the free-text responses from survey 2 that respondents felt were missing from the list of 40 questions were also taken forward.

### Consensus meeting: agreeing final list

**Fig. 1:**
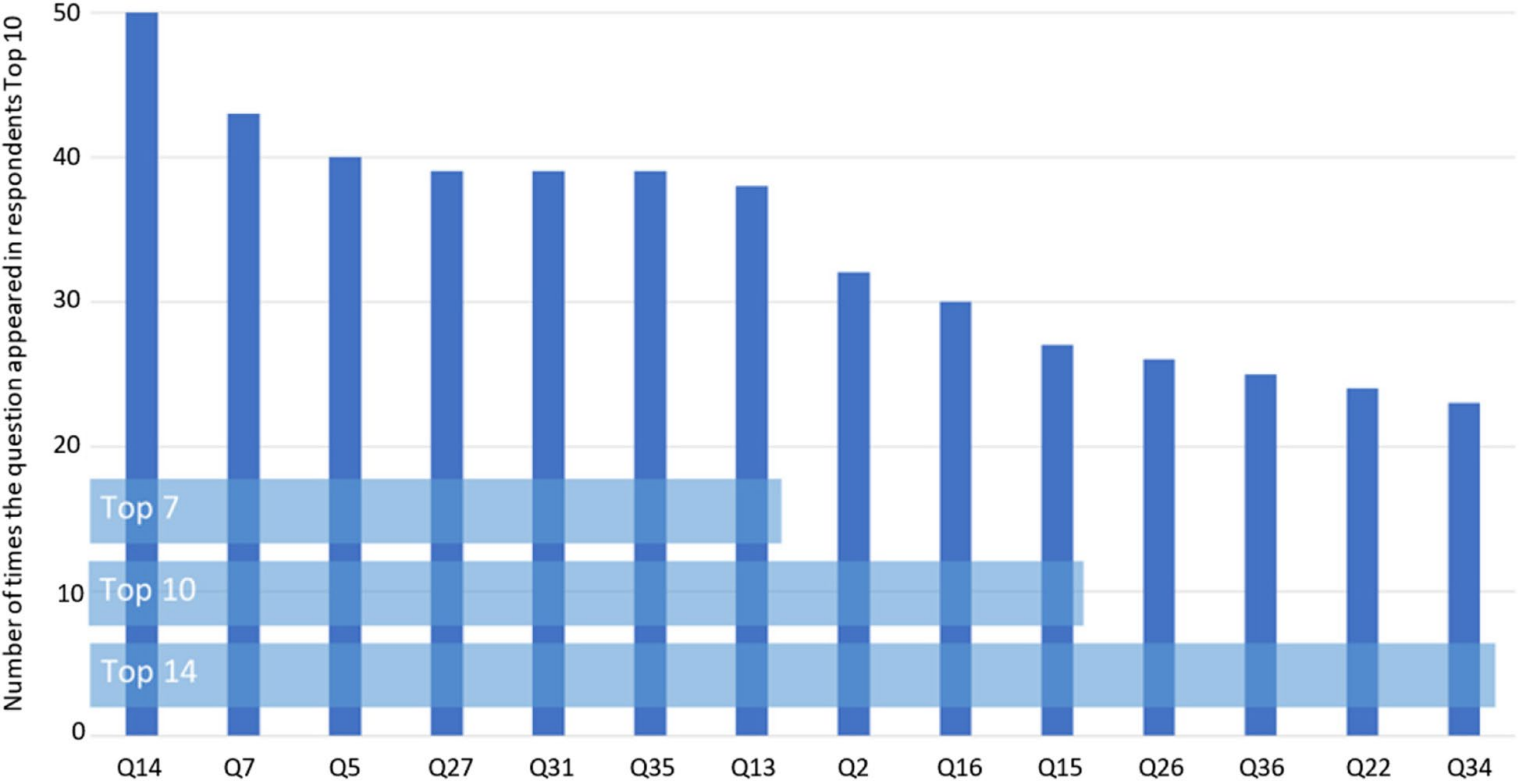
Frequency of questions appearing in the top 10. Question numbers relate to the order of questions shown in Additional file 2

The consensus meeting consisted of 13 stakeholders comprising 11 trialists and two data providers. The consensus meeting started with a presentation of the results from both surveys, ending with the mostly highly ranked 14 questions brought forward and three additional questions. Discussion on the interpretation and scope of the questions moved the three additional questions under the scope of ‘already asked’ questions. There was a clear cut-off at seven questions when looking at the frequency of questions being ranked (Fig. 1), and through Mentimeter®, a vote was also taken on how many questions to ultimately include. The final list of seven was agreed upon through a majority consensus (60% of the votes). Through the consensus meeting and subsequently via email, the wording of these seven questions was edited slightly to improve consistency and clarity. These finalised questions are shown in Table 3 (full rankings available in Additional file 3) and available as an infographic (in order of the trial lifecycle) on the study website at https://www.cardiff.ac.uk/centre-for-trials-research/research/studies-and-trials/view/comorant-uk and available in Additional file 4.

**Table 3:** Top seven questions prioritised

	**Question**	**Domain**
1	How can routinely collected data flow (approval through to data provision) from all providers of data be expedited for analysis?	**Trial open:** data access and receipt
2	When is it more efficient, considering trial design, costs, time and environment, to use routinely collected datasets compared to bespoke data collection?	**Trial design:** data collection method
3	How can approvals at trial set-up be streamlined across regulatory and data provider applications?	**Trial set-up:** regulatory approvals
4	How should the trials community decide when routinely collected data for outcomes is of sufficient quality and utility to replace bespoke data collection?	**Trial design:** outcome selection
5	What causes inconsistencies in routinely collected data across sources and how can these be identified, managed and reconciled for key trial outcomes (e.g. fact and date of death)?	**Trial data:** quality
6	Why are data missing in routinely collected datasets (person and individual data fields) and how should this inform methods for managing missing data?	**Trial data:** analysis
7	What are the best methods to communicate and build trust with trial participants (and the public) about how their routinely collected data will be used?	**Patient and Public Involvement:** communication

## Discussion

### Summary of findings

Through a three-step Delphi, this study systematically identified and then ranked the remaining research questions and uncertainties related to the use of RCD in trials, from the perspective of relevant stakeholders in the UK. Seven questions were prioritised and agreed upon as the top questions within the following domains: trial design, Patient and Public Involvement, trial set-up, trial open and trial data. These questions address both evidence gaps (requiring further methodological research) and implementation gaps (requiring training and/or service re-organisation). These top questions have been published as a freely available infographic.

### Strengths and weaknesses

The numbers of responses for the two surveys were 66 and 88. These are comparable to other recent Delphi studies with similar stakeholder groups or scope (trials using RCD), for example, CONSORT-ROUTINE which received *n* = 92 and *n* = 77 [11]. However, in comparison with the James Lind Alliance (JLA) Priority Setting Partnership (PSP) studies priority I on recruitment (*n* = 790) and priority II on retention (*n* = 456) [6, 12], these response rates are considerably lower. One consideration is the scope of our study is considerably narrower at this stage whereas recruitment and retention are applicable to all trials. In contrast, only a small number of trials (fewer than 5%) are estimated to have been accessing RCD in the UK [13], and therefore, expertise and opportunity to contribute to the scope of this study will be reduced compared to these two JLA PSPs. There is also potential for “consensus project fatigue” among the trials’ community with a rapid increase in the number of such priority-setting exercises in recent years.

Although all stakeholder groups were represented in both surveys, our sample’s composition was dominated by trialists despite our broad engagement strategy. Trialists will, however, have the most experience in all stages of the trial lifecycle (data acquisition to analysis and interpretation) and therefore inherently have more to input in such a focussed Delphi study. Two-thirds of respondents of the second survey either did not contribute to the first survey or were unsure if they had done so and were provided with the opportunity to add in any questions at the end of the survey they considered were missing from the list of 40 questions presented. Only three questions emerged which were subsequently re-categorised during the consensus meeting within one of the 40 questions. This is an indication that additional input from stakeholders may not have yielded substantially different results to those reported here. It is acknowledged that the method of collection prevented a calculation of a response rate between surveys 1 and 2. In addition, we had representation from stakeholders across all four countries in the UK, at all stages of academic career, with expertise in using different types of data sources.

The first survey focussed on remaining questions that were methodological in nature. A definition of *methodological* (the study of methods with a view to improving clinical trials) was provided at the start of this survey. However, a considerable proportion of responses related to operational or implementation challenges require new or clearer guidance rather than new methodology work. Through discussion with the wider study team, it was agreed that all questions should be taken forward in the second survey as some that appeared operational may indeed be addressed via methodological research. This, combined with the additional subjectivity of such categorisation, meant that in practice, it was felt inappropriate to separate them into two groups.

This study focused on UK stakeholders as a starting point, and the findings will only reflect the methodological questions from a UK standpoint. It is important to recognise that some of the key questions are likely to reflect structures and data that vary by country but that some of the short and long-list of challenges are likely to have some relevance to other countries, which may represent scope for future work.

### Implications

These prioritised questions require input from all stakeholders included in this study and beyond. Investment in infrastructure supporting RCD needs to be accompanied by a commitment to understanding the best methods and best practices for the use of RCD in trials. Data providers and regulators in particular will play a large role in addressing the two questions related to streamlining approvals across organisations and expediting timelines for data acquisition. These rely on working together to adapt their organisational processes and/or streamlining across organisations. Funders will continue to be crucial in supporting methodological projects aiming to address many of these questions. Recognition that we need to better understand these questions for the greatest benefit to patients, care teams and the public is gaining traction. Health Data Research (HDR) UK has recently funded a number of time-limited projects that will contribute to answering some of these seven questions and will be well placed to take many of these remaining questions forward over the coming years along with other key stakeholders such as the MRC-NIHR Trials Methodology Research Partnership. Members of the public and public contributors on trials will have particular significance in contributing to communication and building trust in trials that use RCD. Finally, trialists will (and already are) push these questions forward and provide input to the data provider and regulator consultations to ensure trials are included in strategic decisions and organisational prioritisation. The MCR-NIHR Trials Methodology Research Partnership plays a crucial role in this.

## Conclusions

This prioritised list of seven questions will inform the direction of future research in this area, and direct efforts should be made to ensure that the benefits in major infrastructure for routinely collected data are achieved and translated. It is important to consider the findings in light of the limitations presented and that the research priorities are dynamic over time (i.e. problems may get solved, and new ones arise), so any research priority exercise will need to be refreshed periodically. Without this and future work to address these questions, the potential societal benefits of using routinely collected data to help answer important clinical questions will not be realised.

## Supplementary Information

**Additional file 1.** Survey 1: Guidance provided and the format the question was asked.

**Additional file 2.** Full list of 40 questions included in survey 2.

**Additional file 3.** Final ranking of all 40 questions.

**Additional file 4.** Infographic. Also, available on study page: https://www.cardiff.ac.uk/centre-for-trials-research/research/studies-and-trials/view/comorant-uk.

## Data Availability

All data generated or analysed during this study are included in this published article and its supplementary information files. This excludes the free-text data collected in survey 1, which may be requested from the corresponding author on reasonable request after appropriate review.

## Abbreviations

RCD: Routinely collected data
EHR: Electronic health records
JLA: James Lind Alliance
PSP: Priority Setting Partnership
