# Supplementary material for: A DELPHI study priority setting the remaining challenges for the use of routinely collected data in trials: COMORANT-UK
Source: Trials. 2023 Mar 30;24:243. doi: 10.1186/s13063-023-07251-x (PMC10064573; doi:10.1186/s13063-023-07251-x)
Supplement: Supplementary file 1 — Additional file 1. Survey 1: Guidance provided and the format the question was asked. [file 13063_2023_7251_MOESM1_ESM.docx]

Additional file 1

**Survey 1: Guidance provided and the format the question was asked**

**How to complete the survey**

We are interested in all of the ongoing challenges and unanswered questions that remain in relation to routinely-collected data and trials. We’d like you to list all challenges/questions, but are particularly interested in ones that are related to issues that can be answered with research methods (‘methodological’ ones) rather than operational (i.e. can be resolved through a change in process). These will be the ones that are taken forward to the second survey.

Please consider all aspects of the study lifecycle when considering what are the remaining unanswered questions and challenges.

**Study lifecycle**


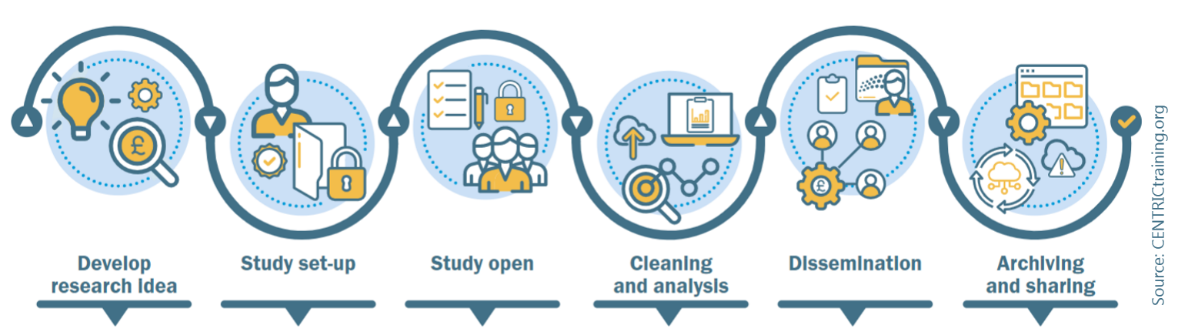


Please list all of the challenges and research questions that you can think of. If you have more than six questions, we will only take forward the first six and the remaining questions will be described in a separate publication**, so please put your most pressing questions first.**

If you can suggest any methods to investigate this, and a reason on why this question is important, please do add this where indicated.

**Questions & Challenges**

Use the spaces to add individually the questions you would like to be included. Please state the question or challenge followed by as much detail as possible - why this is important and any suggested methods. **You do not need to provide 6 responses**, please leave any remaining boxes blank and move to the next page to submit your response.

**Question or challenge 1**

**Question or challenge 2**

**Question or challenge 3**

**Question or challenge 4**

**Question or challenge 5**

**Question or challenge 6**

**Do you have more? We’d love to hear them - please add in the box below!**
