# Supplementary material for: A DELPHI study priority setting the remaining challenges for the use of routinely collected data in trials: COMORANT-UK
Source: Trials. 2023 Mar 30;24:243. doi: 10.1186/s13063-023-07251-x (PMC10064573; doi:10.1186/s13063-023-07251-x)
Supplement: Supplementary file 2 — Additional file 2. Full list of 40 questions included in survey 2. [file 13063_2023_7251_MOESM2_ESM.docx]

Additional file 2

**Full list of 40 questions included in survey 2**

| 1 | What are the best costing models when planning routinely-collected data use in trials? |
| --- | --- |
| 2 | What standardised participant information and consent wording for trials linking to routinely-collected data would be acceptable to all data providers, now and in the future? |
| 3 | What information is required for Ethics / CAG (Confidentiality Advisory Group) regarding routinely-collected data in trials? |
| 4 | What approvals (local and over-arching) are required to access routinely-collected data from multiple sites/organisations? |
| 5 | How can approvals be streamlined across regulatory and data provider applications? |
| 6 | Will regulators accept routinely-collected data within a clinical trial? And if so, what do we need to evidence? |
| 7 | When is it more efficient, considering costs, time and environment, to use routinely-collected datasets compared to traditional trial data collection (e.g. via Case Report Form)? |
| 8 | How can routinely-collected data in trials support criteria for efficacy, effectiveness and safety monitoring? |
| 9 | How accurate are screening approaches to identify eligible patients based on routinely-collected data? |
| 10 | How does the use of routinely-collected data for trial outcomes affect recruitment? |
| 11 | How can blinding be maintained when using registry data? |
| 12 | How can trial participants have sufficient understanding about how their routinely-collected data is used and protected for use in research? Including routinely-collected data being used by industry and private sectors. |
| 13 | What is the best method to communicate and build trust with participants (and the public) about how their routinely-collected data will be used? |
| 14 | How can routinely-collected data access from all providers be expedited to allow timely analysis of outcomes? |
| 15 | How can routinely-collected data related to safety and adverse events be made available within required timescales? |
| 16 | How can data providers align to enable routinely-collected data access for cross-nation and UK wide trials? |
| 17 | Where can trialists access information on what routinely-collected data are available for specific clinical areas and how to access those data? |
| 18 | How best should sample routinely-collected datasets be used for deciding the datasets and variables relevant for a trial? |
| 19 | Where can trial teams find information on the utility of specific variables within routinely-collected datasets (e.g. providing a rate of completion of the variables)? |
| 20 | What diagnostic position must eligibility, outcome or adverse event codes occur in to identify participants or outcomes with sufficient accuracy? |
| 21 | How can events (e.g. diagnoses, prescriptions) that may be longstanding, new, or recurring be appropriately recorded and extracted? |
| 22 | How can the knowledge of how routinely-collected data (including codes) are recorded be translated/communicated for use by those receiving and analysing the data? |
| 23 | How can the trial community assess impact of planned changes to routinely-collected data recording (even if no clinical impact)? |
| 24 | What are the implications of focusing on “complete”, “up to standard” or ‘good’ routinely-collected data on inclusivity? |
| 25 | How can multiple datasets be accurately and demonstrably linked? |
| 26 | How can we develop methods to enrich datasets through data linkage (e.g. linking educational datasets with primary care data)? |
| 27 | How should the trials community decide when routinely-collected data for outcomes is of sufficient quality and utility to replace bespoke data collection? |
| 28 | How can SWATs (Studies Within a Trial) be used to address routinely-collected data quality concerns? How generalisable are they? |
| 29 | Are national routinely-collected data returns (minimum datasets) sufficiently granular for trials research? |
| 30 | Are gender and sex data collected accurately in routinely-collected datasets? And do we know the difference? |
| 31 | What causes inconsistencies in routinely-collected data across sources and how can these be identified, managed and reconciled for key trial outcomes (e.g. fact and date of death)? |
| 32 | How does data quality for similar outcomes vary by data provider including between UK nations? |
| 33 | How can trial teams ensure the routinely-collected data requested and reported reflects the same understanding as why it was recorded? |
| 34 | How should data providers engage with the staff recording the routinely-collected data to improve data quality and optimise for trials research? |
| 35 | How can the trials community understand reasons for missingness in routinely-collected datasets and how should this determine methods for managing missing data? |
| 36 | What are the best and most cost-effective methods for retaining routinely-collected data at the end of the trial whilst aligning with regulatory and data provider requirements? |
| 37 | Where do I store the routinely-collected data throughout the trial lifecycle (analysis, archive, onward sharing) to be safe and acceptable by data providers, regulators, funders and participants? |
| 38 | How does routinely-collected data ownership affect data retention and requirements for open access? |
| 39 | What is the most cost-effective method for onward data sharing of routinely-collected data? |
| 40 | What are the key considerations and approaches when anonymising routinely-collected data used in trial? |
