# Supplementary material for: A DELPHI study priority setting the remaining challenges for the use of routinely collected data in trials: COMORANT-UK
Source: Trials. 2023 Mar 30;24:243. doi: 10.1186/s13063-023-07251-x (PMC10064573; doi:10.1186/s13063-023-07251-x)

Additional file 4: Infographic. Also, available on study page: <https://www.cardiff.ac.uk/centre-for-trials-research/research/studies-and-trials/view/comorant-uk>
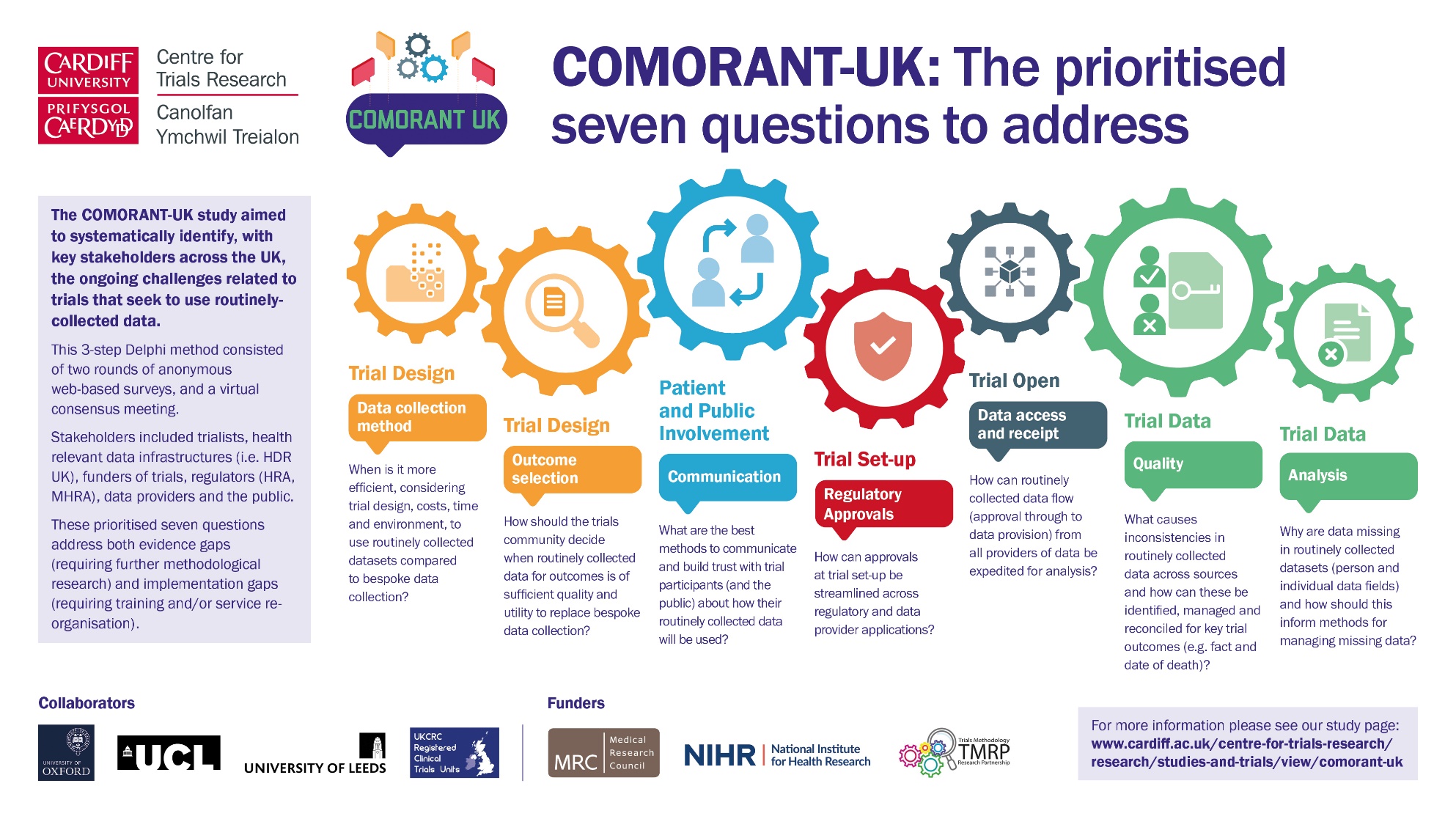

Supplement: Supplementary file 4 — Additional file 4. Infographic. Also, available on study page: https://www.cardiff.ac.uk/centre-for-trials-research/research/studies-and-trials/view/comorant-uk. [file 13063_2023_7251_MOESM4_ESM.docx]
